# Chromatin mutations in pediatric high grade gliomas

**DOI:** 10.3389/fonc.2022.1104129

**Published:** 2023-01-06

**Authors:** Hsiao P. J. Voon, Lee H. Wong

**Affiliations:** Department of Biochemistry and Molecular Biology, Biomedicine Discovery Institute, Monash University, Clayton, VIC, Australia

**Keywords:** histone H3.3, H3.3 K27M, DMG = diffuse midline glioma, H3.3 G34R/V, ATRX, pediatric gliomas, KDM4, alternative lengthening of telomeres (ALT)

## Abstract

Pediatric high grade gliomas (HGG) are lethal tumors which are currently untreatable. A number of recent studies have provided much needed insights into the mutations and mechanisms which drive oncogenesis in pediatric HGGs. It is now clear that mutations in chromatin proteins, particularly H3.3 and its associated chaperone complex (ATRX), are a hallmark feature of pediatric HGGs. We review the current literature on the normal roles of the ATRX/H3.3 complex and how these functions are disrupted by oncogenic mutations. We discuss the current clinical trials and pre-clinical models that target chromatin and DNA, and how these agents fit into the ATRX/H3.3 mutation model. As chromatin mutations are a relatively new discovery in pediatric HGGs, developing clear mechanistic insights are a key step to improving therapies for these tumors.

## Introduction

Gliomas are the most common form of primary brain tumors and are currently lethal in both children and adults. Over the past decade, a number of large-scale genome sequencing studies have identified key mutations which drive oncogenesis in these tumors (1–4). From these studies, it has become increasingly clear that adult and pediatric gliomas are distinct biological entities with specific mutational profiles. These differences are now officially recognized in the latest 2021 WHO classification of CNS tumors (5). One of the clearest features which distinguishes pediatric from adult gliomas, are the high rates of mutations in chromatin-related proteins in pediatric tumors (6). Specifically, mutations in histone genes have been officially designated as diagnostic subgroups of pediatric-type diffuse high-grade gliomas: diffuse midline glioma, H3K27-altered; diffuse hemispheric glioma, H3 G34-mutant (5). The overwhelming majority of these histone point mutations occur on the histone variant H3.3 (83% of K27M mutations, 100% of G34R/V mutations) (4), with rare mutations in canonical histone H3.1.

In addition, H3.3 mutations in pediatric gliomas frequently occur in conjunction with inactivating mutations in ATRX (20% H3.3 K27M, >90% of H3.3 G34R) (4). ATRX is a SNF2 helicase/ATPase (7) that partners with DAXX (8, 9) to form an H3.3 chaperone complex (10, 11). Taken together, this suggests that H3.3 and its ATRX chaperone complex are core contributors to oncogenesis in pediatric gliomas. Furthermore, inactivating mutations in ATRX are also found in conjunction with point mutations in isocitrate dehydrogenase (IDH) in adult-type diffuse gliomas (>86%) (12, 13), recently designated as “astrocytoma, IDH-mutant” (5). Mutations in IDH1/2 (mIDH) generate an oncometabolite which inhibits a range of chromatin modifiers and, similar to H3.3 mutations, severely disrupt chromatin profiles. IDH mutations are most common in adolescents (14) and younger adults (<55 years) (15), possibly indicating a graded continuum between these chromatin mutations and age-of-onset. This review will focus on the current understanding of these glioma-associated chromatin mutations which affect younger age groups.

## Histone H3.3

**Figure 1:**
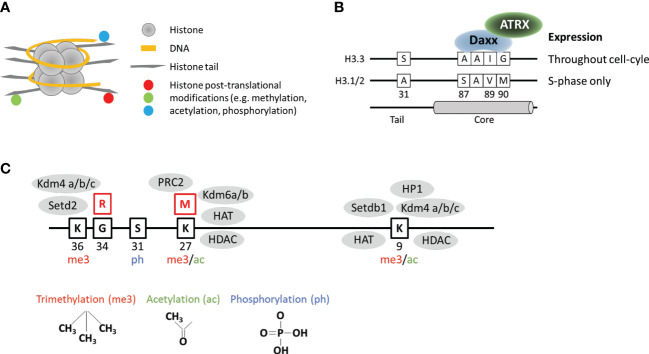
Key structural and functional features of histone H3.3. **(A)** DNA wrapped around nucleosome comprised of histones arranged into an octamer configuration with protruding tails. **(B)** Key features which distinguish histone variant H3.3 from canonical H3.1/2. **(C)** Selected amino acid residues on the H3.3 tail which are regulated by post-translational modifications. Red boxes show the position of frequently mutated residues. Grey ovals represent examples of epigenetic readers, writers, and erasers known to interact with mutated and surrounding residues.

Histones are the protein component of nucleosomes, which form the basic repeated structural unit of chromosomes. Each nucleosome consists of ~146 bp of DNA wrapped around a histone octamer comprised of two units each of histone H2A, H2B, H3, and H4 (**Figure 1A**). The majority of nucleosomes are comprised of “canonical” histones such as histone H3.1/2 which are encoded by 13 genes in the human genome (16). Histone H3.1/2 are synthesized only during S-phase of the cell cycle and are rapidly assembled behind the DNA replication fork (16). Unlike canonical replication-dependent H3.1/2, histone variant H3.3 is expressed throughout the cell-cycle in a replication-independent manner (17) and is thought to replace histones which are displaced outside of S-phase (18).

Histone H3.3 is encoded by two genes in the genome (*H3F3A* and *H3F3B*) and differs from canonical H3.1/2 at 5/4 amino acid residues respectively at positions 31, 87, 89, 90, with an additional difference at 96 between H3.3 and H3.1. The so-called “AAIG” motif at positions 87, 89, and 90 determine the interactions with chaperone assembly complexes (11) (**Figure 1B**). The combination of replication-independent synthesis and specific chaperones means that H3.3 has a genome localization pattern and function, which is unique and distinct from canonical H3.1/2. As H3.1/2 are linked to DNA replication, the canonical histones are uniformly distributed across the genome in the wake of replicating DNA polymerase. In contrast, H3.3 is most often associated with the promoters of active genes where it replaces histones which have been displaced by the passage of RNA polymerase (18–20). In addition, H3.3 is also associated with unusual chromatin environments such as telomeres (10, 20, 21), ERV repeats (22, 23), and imprinted genes in mouse embryonic stem (ES) cells (22); the V_H_ locus which undergoes V(D)J recombination (24); primordial germ cells (25), and complete remodeling of the paternal genome post-fertilization (26, 27).

As a result, H3.3 has been associated with diverse functions including fertility, embryogenesis, maintenance of stem cell states, and execution of differentiation programs (28). It is not entire clear why H3.3 is uniquely important for maintaining or altering chromatin states but it is evident that despite the high degree of sequence similarity, H3.3 is functionally distinct from canonical H3.1/2. The unexpected discovery of oncogenic H3.3 point mutations further emphasized this difference and shifted our collective understanding of chromatin biology and associated mutations in cancer. The first studies in this area discovered heterozygous substitution mutations at position 27 (lysine) and 34 (glycine) exclusively on *H3F3A* of H3.3 (2, 3), as well as a minority on K27M (lysine to methionine) mutation on HIST1H3B (H3.1). Interestingly, the H3.3 K27M mutations appear to be distinct from H3.1 K27M counterparts as they are associated with different secondary mutations (29), age-of-onset (29), and chromatin and gene expression profiles (30–32). It is therefore highly likely that the H3.3 mutations in pediatric gliomas are functionally significant in a manner that is related to the normal endogenous functions of H3.3.

## H3.3 K27M mutations

Of these histone mutations, the H3.3 K27M mutations are the most common and therefore also the most well-studied. Early studies reported H3.3 K27M mutation rates of >90% in tumors which were then classified as Diffuse Intrinsic Pontine Gliomas (DIPG). The WHO CNS tumors classification schema has since been updated and H3.3 K27M is now considered a defining feature of a class of pediatric high-grade gliomas dubbed “diffuse midline glioma, H3 K27-altered” (5). H3.3 K27M mutations most often arise in midline structures including the thalamus, pons and brainstem, with a median age of diagnosis of around 9-10 years (4). The nature and location of these tumors severely limits treatment options and the 2-year survival rate is <10%, with a median survival time of 11 months (4). The H3.1 K27M mutations are specific to the pons and occur in a younger age group (median 5 years) and are associated with a median survival time of 15 months (4).

Early studies suggested that the H3.3 K27M mutation was acting primarily through inhibition of the Polycomb Repressive Complex 2 (PRC2) (33, 34), a methyltransferase which mediates trimethylation of lysine 27 (35). The H3K27me3 modification is primarily associated with the promoters of silenced genes and is important for regulation of gene expression, particularly through differentiation (35, 36). The H3.3 K27M mutation consistently triggers the global loss of H3K27me3 and *in vitro* studies indicated that the K27M mutation was capable of binding and inhibiting PRC2 (33, 34). However, direct interactions between H3.3 K27M and PRC2 have proven difficult to detect *in vivo* (37, 38) and these proteins have distinct localization profiles (31). PRC2 primarily localizes to promoters of silenced genes (36) while histone H3.3 is primarily associated with regions of high nucleosome turnover, notably the promoters of active genes (20).

In addition, it has become increasing clear that a broad range of chromatin modifications are disrupted in the presence of this mutation. As well as reductions in H3K27me3, the K27M mutation is also associated with reduced DNA methylation (hypomethylation) across the genome (39), increased H3K27ac (38, 40, 41), reduced H3K36me2 (42, 43), and increased H3K9ac and H3K4me3 (44). As there is no consensus on proteins which interact directly with the K27M mutation, the primary chromatin alterations associated with this mutation are currently unknown. Given the conflicting studies and the diverse range of chromatin alterations, it is possible that there are multiple interacting partners which are disrupted by the K27M histone tail mutation. The H3K27 residue is important for gene regulation and is a target for both post-translational methylation and acetylation, which regulate silencing and activation respectively (45) (**Figure 1C**). The substitution mutation could potentially affect the activity of K27 methyl- and acetyl- transferases as well as the demethylases and deacetylases. It is also possible that chromatin readers, writers and erasers which target neighboring residues could be disrupted by the K27 substitution (**Figure 1C**). Furthermore, as the H3.3 K27M mutation appears to be distinct from the H3.1 mutations, there may be histone-specific interactors which have thus far been overlooked.

## H3.3 G34R mutations

A second frequent histone mutation in pediatric gliomas is a substitution of the glycine residue at position 34, most often to arginine (G34R, 94%) and less frequently to valine (G34V, 6%) (4). The G34R/V mutations occur exclusively on histone H3.3 (3, 6, 46) and frequently overlap with inactivating mutations in ATRX and TP53 (90%) (4). The H3.3 G34R/V mutations are most often found in high grade gliomas localized to the cerebral hemispheres with a median age of diagnosis of 15 years and a median survival time of 17-18 months (4, 47). Additional G34 substitutions have also been reported in giant cell tumor of bone (G34W/L/R/V/M) (48) and osteosarcomas (G34W/R) (49).

Unlike the K27 residue, G34 is not a direct target for post-translational modifications but is located close to the K36 residue which is trimethylated (K36me3) (**Figure 1C**). H3K36me3 predominantly localizes to the bodies of active genes and is associated with elongating RNA polymerase II (50). This modification is thought to suppress cryptic initiation of transcription (51) by suppressing histone turnover within gene bodies (52). The substitution of a small glycine to a bulky arginine residue has been suggested to inhibit the activity of the H3K36 methyltransferase (SETD2) (53) and the K9/K36 demethylase (KDM4) (54) (**Figure 1C**). Inhibition of SETD2 reportedly occurs *in cis* and would therefore only affect the K36 residue on the mutated histone (53). In contrast, the chromatin alterations (increased H3K9me3 and H3K36me3) associated with inhibition of KDM4 were observed across the genome which is consistent with a dominant negative effect expected from these mutations, although the specific mechanism remains unclear (54). In addition to H3K36me3 and H3K9me3, the H3.3 G34R mutations have also been associated with altered patterns of H3K27me3 (53), and DNA methylation (55). Furthermore, the G34R substitution also interferes with reader proteins such as ZMYND11 (56) and ZMNYD8 (57) which bind this region of the histone tail, and has been associated with altered splicing (58).

As for the K27M substitutions, there is no single clear unifying model to account for the primary defects and downstream effects of the H3.3 G34 substitution mutation. Indeed, the current observations suggest that G34 mutations could affect multiple chromatin pathways simultaneously, which is likely to also be the case for the K27M mutation. Given that the G34 substitutions occur exclusively on histone H3.3, it is likely that the oncogenic mechanism is specific to this variant histone. In support of this, the H3.3 G34R mutation often overlaps with inactivating mutations in ATRX which is part of an H3.3 chaperone complex.

## ATRX mutations

ATRX is a chromatin remodeler which forms a complex with DAXX, an H3.3-specific chaperone, to deposit H3.3 and maintain H3K9me3 heterochromatin silencing at repetitive DNA. This complex is frequently mutated across a range of cancers, and mutations are strongly associated with activation of a telomere maintenance pathway known as Alternative Lengthening of Telomeres (ALT) (59). A recent study found around 17% of all pediatric high grade gliomas (pHGG) have inactivating mutations in ATRX (4). Of the ATRX-mutated HGGs, 33% overlap with H3.3 G34R/V and 50% overlap with H3.3 K27M mutations (4). There is no overlap between ATRX and H3.1 K27M mutations, which are instead associated with mutations in ACVR1 (4, 29, 60). These findings strongly suggest that histone H3.3 plays an important oncogenic role in pHGG.

Unlike the histone mutations, ATRX-mutated pHGGs show no particular regional or temporal specificity. Indeed, ATRX mutations extend into young adulthood and occur at high frequency in adult low grade gliomas (13, 61), as well as other cancers such as pancreatic neuroendocrine tumors (62), pediatric osteosarcomas (63), sarcomas (64–66), pheochromocytomas and paragangliomas (67, 68). These mutations are strongly associated with the ALT telomere maintenance pathway (59), most likely due to disruption of H3.3 incorporation at telomeres. Puzzlingly, patients with ATR-X syndrome who inherit germline mutations in ATRX, do not appear to have an increased risk of cancer (69). This suggests that ATRX mutations are necessary but not sufficient to activate ALT, and additional mutations are likely required.

Given that ATRX mutations frequently co-occur with H3.3 mutations in pediatric gliomas, H3.3 mutations are good candidates for potential partners in ALT activation. Consistent with this, H3.3 G34R has been reported to consistently activate ALT when combined with inactivating mutations in ATRX, TP53 and telomerase (TERT) in mouse ES cells (70). This was attributed to inhibition of the H3 K9/K36 demethylase, KDM4B, by the H3.3 G34R mutation. It appears that the loss of telomeric H3.3 (ATRX KO) combined with increased H3K9me3 through inhibition of KDM4B, results in a chromatin environment that supports the formation of ALT-associated PML bodies (APBs) which are essential for telomere maintenance (70). PML bodies are naturally occurring phase-separated nuclear condensates (71) which become abnormally large and localise to telomeres in ALT-positive cancers (72). One of the main drivers of phase-separation is heterochromatin protein 1 (HP1α) (73), a protein which binds to the H3K9me3 modification (74). It seems that inhibition of the KDM4B demethylase results in increased H3K9me3 to facilitate phase-separation and APB formation.

## IDH1/2 mutations

Interestingly, point mutations in a citric-acid cycle enzyme, isocitrate dehydrogenase (IDH1/2), are also known to inhibit this family of lysine demethylases (75). IDH mutations are relatively rare (~6%) in pediatric HGGs and tend to occur in the forebrain of older patients with a median age of 17 years (4). However, IDH mutations occur at very high frequency (~80%) in adult low-grade gliomas (aLGG, WHO grade II and III) (13) and tend to be associated with younger cohorts (76). The majority (52%) of IDH-mutated aLGGs overlap with ATRX/TP53 inactivating mutations while the remainder are predominantly oligodendrogliomas which co-occur with 1p/19q co-deletion mutations (13). The high frequency overlap between IDH and ATRX mutations is reminiscent of the H3.3/ATRX mutations in pediatric high-grade gliomas, and hints at similarities between the IDH and H3.3 mutations.

The IDH1/2 enzymes catalyze the oxidative decarboxylation of isocitrate to 2-oxoglutarate/α-ketoglutarate (2-OG/α-KG), which is a key reaction in the citric acid cycle. In addition, α-KG serves as a cosubstrate for αKG-dependent dioxygenases, which include the TET family of 5-methylcytosine hydroxylases and histone lysine demethylases such as the KDM4 family of enzymes (75). Oncogenic mutations in IDH most often occur as heterozygous, dominant negative point mutations at the active site of IDH1 (R132) or IDH2 (R172) (77). These mutations convert αKG to R(-)-2-hydroxyglutarate (2HG), an oncometabolite that inhibits αKG-dependent dioxygenases, including the KDM4 family that is affected by H3.3 G34R mutations (75, 78).

Inhibition of histone lysine demethylases could therefore be a common factor which unites histone H3.3 and IDH mutations. Consistent with this, both mutations frequently co-occur with ATRX inactivation in gliomas, and both H3.3 G34R and IDH1 R132H have been shown to promote ALT when combined with inactivation of ATRX/TP53 and telomerase (70). In addition, much like the histone mutations, IDH1/2 mutated gliomas also exhibit broad disruptions in chromatin modifications including DNA methylation (79) and histone methylation (H3K9me2, H3K27me2, H3K79me2) (75), which ultimately results in a failure in differentiation (78). At present, the common theme across the H3.3/IDH mutations appears to be inhibition of demethylases, coupled to defects in H3.3 either directly or through inactivation of ATRX, leading to widespread chromatin alterations that block differentiation. Although the specific pathways which are most affected by H3.3/IDH mutations are currently under investigation, it is clear that chromatin disruption is a common feature across these gliomas. This would potentially render these cancers vulnerable to DNA damaging agents and epigenetic drugs regardless of specific targets, and a number of these agents are currently being trialled.

## Clinical trials

The current management of pediatric gliomas typically includes a combination of surgical resection and radiotherapy. However, due to the location and infiltrative nature of high grade gliomas, complete resection is often not possible and treatment is usually palliative. Chemotherapy has proven ineffective for pediatric high-grade gliomas thus far. In addition to the universal issues of efficacy, selectivity and acceptable adverse side-effects, effective drugs must also be capable of crossing the blood brain barrier. A range of potential candidates are currently being trialed but clear leads or principles have yet to emerge.

Developing a clearer understanding of mutations and molecular mechanisms should provide some guidance on the best strategies to trial, with the ultimate goal of developing targeted and specific therapies. In accordance with this, there are a number of ongoing trials which attempt to target the K27M peptide specifically through a neoantigen peptide (NCT04749641) or a peptide vaccine in combination with the PD-1 inhibitor, nivolumab (NCT02960230) (80). In addition, the IDH mutations are also an attractive target and trials are somewhat more mature as the IDH mutations are more common, occurring at high frequency in adult low grade gliomas as well as acute myeloid leukemia (AML). Inhibition of mutant IDH2 with enasidenib (81) or IDH1 with ivosidenib (82) is effective at treating IDH-mutated AML providing clinical evidence that inhibition of the mutant enzyme is beneficial. Phase I trials in low grade gliomas found that ivosidenib was well tolerated and reduced tumor volume (83). Trials with vorasidenib, a mutant IDH1/2 inhibitor with improved blood brain penetration, was similarly well tolerated and showed preliminary antitumor activity (84). While IDH inhibitors have not yet been trialed specifically in IDH mutated pediatric high grade gliomas, positive outcomes in adult gliomas would likely be translated to the pediatric cohort.

In addition, there are trials underway for inhibiting EZH2 (PRC2) with tazemetostat (NCT03155620), and the polycomb protein BMI1 with PTC596 (NCT03605550) (85). Although it is not entirely clear that K27M acts through PRC2, it is clear that both the histone and IDH mutations cause widespread disruptions across the genome regardless of the specific mechanisms. Therefore, it is possible that chromatin and genome targeting agents would further exacerbate this phenotype and trigger cell death. Indeed, the two strategies which have some proven efficacy in gliomas both rely on DNA damage. Radiation is a potent DNA damaging agent and temozolomide which is used to treat adult gliomas, is an alkylating agent that damages DNA by methylating purine (guanine, adenine) bases. Although efficacy is obviously limited and treatment only extends lifespan by months, trials are currently underway to test re-radiation and combinations with other drugs including chromatin and DNA damaging agents [reviewed in (80)].

A number of chromatin, epigenetic, and DNA damaging drugs are routinely used in chemotherapy regimes across different cancers but none have proven effective as single agents in pediatric high-grade gliomas. As a result, most current trials involve testing these drugs in combinations with other agents. The most frequently used class of chromatin and epigenetic drugs are the histone deacetylase inhibitors (HDACi) which include panobinostat (NCT02717455, NCT04341311) and a nanoparticle formulation, MTX110 (NCT03566199, NCT04264143), fimepinostat (NCT02909777, NCT03893487), and vorinostat (NCT02420613, NCT01189266). Drugs that target the genome include agents such as nucleoside analogues (gemcitabine, NCT02992015), topoisomerase inhibitors (etoposide NCT04049669; irinotecan NCT01837862; and topotecan NCT03709680), and alkylating agents (temozolomide NCT03709680, NCT04049669, NCT03243461; lomustine NCT04049669; carboplatin NCT01837862). In addition, inhibition of DNA repair pathways using poly ADP-ribose polymerase (PARP) inhibitors have been hypothesized to complement IDH inhibition and DNA alkylation by blocking the break-excision repair pathway and a number of these are now in trials (BGB-290 NCT03749187; olaparib NCT03233204; veliparib NCT03581292). It will be interesting to see if any of these trials yield positive results.

## Pre-clinical models

It should be noted that the majority of the compounds which are currently in clinical trials are already used in the treatment of other cancers. However, there is potential for developing entirely novel compounds with improved specificity, and this process would be greatly expedited by pre-clinical models which accurately reflect pediatric HGGs. Pre-clinical models can be roughly divided into three groups each with their own advantages and shortcomings: patient-derived cell lines, patient-derived xenograft (PDX) animal models, and animal models with clinically-relevant endogenous mutations.

While patient-derived models should theoretically mirror individual tumors (86), it is inevitable that *in vitro* or *ex vivo* cell culture and transplantation will induce and/or select for alterations in the tumor cells relative to *in situ* counterparts. Amongst the primary concerns are tumor heterogeneity and clonal selection. It is impossible to capture the complex endogenous environment of tumors and every manipulation from cell culture to transplantation applies artificial selective pressure which alters the morphology and clonality of the patient-derived cells (87, 88). While PDX models of pediatric gliomas have been established (89, 90), these models have only undergone limited molecular characterization and it is not currently clear how well these systems reflect endogenous tumors.

One alternative to patient-derived models is the creation of engineered mouse models with clinically relevant mutations which develop equivalent tumors. Given the high frequency of H3.3 mutations in pediatric HGGs, it is very clear that these mutations are oncogenic drivers yet it seems that they are not sufficient to drive tumorigenesis in mouse models (33, 91). Constitutive expression of H3.3 K27M is embryonically lethal (92) and expression must be limited to neural lineages. Expression of H3.3 K27M in isolation does not result in tumor formation (91, 92) but adding a TP53 mutation induces HGG formation at low frequencies (91, 92). The frequency of HGG formation can be boosted with the addition of PDGFRA (91, 92) but these mutations rarely co-occur in patient tumors. The difficulties in establishing model systems has been attributed to restricted developmental stages and cell lineages which are vulnerable to H3.3 mutations (93). Further research into mutations and mechanisms may improve these mouse models in the future however it still remains to be seen if these models can accurately reflect the human tumors.

No model system can completely capture the complexity of patient tumors and all models will suffer from unavoidable pitfalls. It is therefore vitally important that multiple model systems are developed in parallel so that potential therapies can be tested across a range of systems. As with all experimental strategies, orthogonal approaches are the gold-standard for maximizing the chances of identifying efficacious agents while minimizing potential for harm.

## Concluding remarks

Given that pediatric high grade gliomas have proven resistant to all interventions, any degree of improvement would be welcome at this stage. Based on the high rates of mutations in chromatin protein and the adverse effects of these mutations on the genome, it is almost certain that genome targeting agents would prove beneficial as part of combinatorial strategies. However, given the non-specific effects of these drugs and the sensitive nature of neural tissues, off-target effects are likely to pose an issue. As is true for most therapies, targeted delivery and boosting specificity will play an important role in improving overall outcomes and developing accurate pre-clinical models will greatly expedite this process. In addition, further studies into the exact molecular mechanisms behind these mutations could help to uncover pathways that can be targeted with greater specificity and efficacy.

## Author contributions

HV and LW contribute to the writing and editing. All authors contributed to the article and approved the submitted version.
